# Multisensory integration mechanisms during aging

**DOI:** 10.3389/fnhum.2013.00863

**Published:** 2013-12-13

**Authors:** Jessica Freiherr, Johan N. Lundström, Ute Habel, Kathrin Reetz

**Affiliations:** ^1^Diagnostic and Interventional Neuroradiology, RWTH Aachen UniversityAachen, Germany; ^2^Department of Clinical Neuroscience, Karolinska InstituteStockholm, Sweden; ^3^Monell Chemical Senses Center, PhiladelphiaPA, USA; ^4^Department of Psychology, University of PennsylvaniaPhiladelphia, PA, USA; ^5^Department of Psychiatry, Psychotherapy, and Psychosomatics, RWTH Aachen UniversityAachen, Germany; ^6^JARA BRAIN – Translational Brain Medicine, RWTH Aachen UniversityAachen, Germany; ^7^Department of Neurology, RWTH Aachen UniversityAachen, Germany; ^8^Institute of Neuroscience and Medicine (INM-4), Research Center Jülich, JülichGermany

**Keywords:** crossmodal sensory integration, cognition, multimodal, aging neuroscience, elderly population, multisensory integration

## Abstract

The rapid demographical shift occurring in our society implies that understanding of healthy aging and age-related diseases is one of our major future challenges. Sensory impairments have an enormous impact on our lives and are closely linked to cognitive functioning. Due to the inherent complexity of sensory perceptions, we are commonly presented with a complex multisensory stimulation and the brain integrates the information from the individual sensory channels into a unique and holistic percept. The cerebral processes involved are essential for our perception of sensory stimuli and becomes especially important during the perception of emotional content. Despite ongoing deterioration of the individual sensory systems during aging, there is evidence for an increase in, or maintenance of, multisensory integration processing in aging individuals. Within this comprehensive literature review on multisensory integration we aim to highlight basic mechanisms and potential compensatory strategies the human brain utilizes to help maintain multisensory integration capabilities during healthy aging to facilitate a broader understanding of age-related pathological conditions. Further our goal was to identify where further research is needed.

## Multisensory integration

Each of our sensory systems provides us with a qualitatively distinct subjective complementary impression of our surrounding, which are of critical importance for perception, cognitive processing and control of action and can occur in a highly automatized manner (Meredith and Stein, 1983, 1985; Stein and Meredith, 1990). However, most of our everyday percepts are conveyed by multiple sensory systems like the olfactory, auditory, visual, gustatory, and tactile system. Our brain has the remarkable ability to integrate even disparate and complex multisensory information into a unique and coherent percept. The computational mechanisms responsible for this integration assure that the signal is amplified and any kind of accompanied noise is filtered out, thereby promoting the saliency of ecologically meaningful events. Input from two sensory channels, in comparison to a single one, increases the likelihood and speed of detecting and correctly identifying events (Gottfried and Dolan, 2003; Dematte et al., 2006, 2009) and also enhances sensory sensitivity (Dalton et al., 2000; Chen and Spence, 2011). It has also been indicated that higher cognitive sensory processing, like pleasantness evaluation of an odor, is enhanced when it is combined with a congruent auditory stimulus (Seo and Hummel, 2011). Overall, numerous results indicate that multisensory integration plays an important role in our daily life by facilitating and improving our perceptual capacities.

In more detail, multisensory integration is governed by four different principles. First, unimodal sensory stimuli need to be applied within a certain temporal sequence and second, spatial concordance is necessary in order to achieve optimal integration results (King and Palmer, 1985; Meredith and Stein, 1986). Thus, sensory stimuli of different modalities have to coincide with regards to time and space in order to be integrated. Third, contextual, semantic congruency, or correspondence, is fundamental for efficient multisensory integration (Spence, 2011). When those preconditions are fulfilled, the sensory stimuli appear as if emanating from the same object. Importantly, multisensory integration processes follow the principle of inverse effectiveness—cross- or multisensory integration is most effective and therefore elicit maximal behavioral enhancements when less intense or weak and ambiguous individual stimuli are applied (Stein and Stanford, 2008).

Initial research on multisensory integration observed the superior colliculus of anesthetized cats as an important part of the neural network involved (Meredith and Stein, 1983, 1985; Stein and Meredith, 1990). Nowadays, it has been demonstrated that the traditional cortical network supporting multisensory integration in humans consists primarily of the superior temporal sulcus (STS) and the intraparietal sulcus (IPS); it has been suggested that the STS is associated with the integration and labeling of object identity, whereas the IPS is involved in a low-level spatial information processing (Calvert, 2001; Stein and Stanford, 2008). Further, the insula is considered a key area for detection of crossmodal coincidence and matching (Calvert, 2001). However, orbital and ventral areas of the frontal cortex and hippocampal areas have also been identified as neuronal correlates of bimodal multisensory integration involving the olfactory and gustatory domains (Gottfried and Dolan, 2003; Small and Prescott, 2005). Those areas might be responsible for attention and memory processing and are involved in novelty detection, congruency assessment, or task difficulty evaluation (Calvert, 2001). Within frontal areas, a dissociation with regard to object identity exists: the orbital network seems to be especially important for the integration of multisensory input related to food items while the ventrolateral prefrontal region mediates the assessment of non-food objects (Price, 2008).

The existing functional imaging research regarding multisensory integration processes can be divided into two topics: multisensory integration of (i) object observation and (ii) emotional perception. For the investigation of multisensory processes during object recognition, combinations of two different modalities—combined visual-auditory stimulations—is the most utilized. A concurrent combination of three different and congruent sensory stimuli has yet to be applied within a neuroimaging setting. However, when analyzing areas of overlap between the three senses of touch, sound, and vision, both the STS and IPS demonstrate a considerable overlap in processing (Bremmer et al., 2001; Beauchamp et al., 2008; Langner et al., 2012). In addition, the left fusiform gyrus (FG) seems to play a putative role within trimodal sensory object manipulation (Kassuba et al., 2011). Most of these studies are, however, based upon a combination of simple stimuli or a conjunction analysis of brain activation related to unimodal sensory stimulation. Multisensory integration mechanisms are of special importance during the perception and processing of emotions. Research from our own and other groups provides evidence for a neural network involving the amygdala, insula, frontal areas, FG, and STS which are responsible for integration of cross- or multisensory information related to emotional perception during stimulation with dynamic stimulus material of different modalities (Ethofer et al., 2006; Kreifelts et al., 2009; Seubert et al., 2010a,b; Klasen et al., 2011, 2012; Muller et al., 2011, 2012; Regenbogen et al., 2012a,b). Although we gained novel insights into multisensory integration processes with regards to object and emotion perception during the last years, a systematic investigation of multisensory integration in relation to differences between age groups and age-related pathologies using functional imaging means is still missing. Since emotional perception accounts for a major part of our everyday wellbeing and multisensory processes are heavily involved in emotional processes we want to draw attention towards a better understanding of multisensory processes and the underlying neural connections during emotional perception. Therefore, the aim of this review is to outline the knowledge about multisensory integration across the lifespan with a special focus on behavioral and neural correlates of multisensory integration during aging and age-related diseases. We further aim to identify areas where further research is needed in order to shed light onto the mechanisms of multisensory integration.

## Multisensory integration during aging

In light of the evolving demographic changes of our society, one important future task for the research communities is to further our understanding of lifelong healthy aging. Aging is a multifactorial and multidimensional process involving physiological, psychological, and social alterations. As part of ongoing degeneration throughout life, there is a progressive deterioration of physical function leading to loss of viability and an increase in vulnerability. Particularly, sensory impairments have an enormous impact on our lives and are closely related to intellectual functioning. Because we experience our environment through multiple sensory systems, which ultimately ensure our everyday safety, quality of life, and social adjustment, it is of interest to understand how multisensory integration processing changes as a function of healthy aging. In facing these challenges, the following question is noteworthy: what age-dependent changes in the neuronal integration of multisensory stimuli occurs in individuals experiencing healthy aging?

Effectiveness of multisensory integration depends upon functioning of the peripheral sensory organs as well as higher cognitive processes. As we age, we experience a decline of function in all our five senses—e.g., visual acuity decreases (Spear, 1993) and auditory thresholds increase (Liu and Yan, 2007). Olfactory capabilities are known to decline during aging as well (Rawson, 2006), however this deterioration can be attributed to a poor medical status and cognitive decline in the elderly (Nordin et al., 2012). Typically, motor speed and executive functions (Falkenstein et al., 2006), as well as working memory and attention control deteriorate as well (Fabiani, 2012). Previous structural neuroimaging studies, including voxel-based morphometry (VBM), deformation field morphometry (DBM), cortical thickness analyses, manual tracing techniques, and diffusion-weighted magnetic resonance imaging (MRI) have given some insights into the complexity of age-related structural brain changes (Sowell et al., 2004; Raz and Rodrigue, 2006; Sala et al., 2012). In a recent review, Hedman et al. (2012) concluded that apart from brain volume increases during childhood and adolescence, a continuous volume decrease of 0.2% per year can be observed, which accelerates to an annual brain volume loss of 0.5% at age 60 and more than 0.5% above the age of 60 years. Thereby, evidence is provided for a volume loss of gray matter and cortical thinning during aging. However, not only structural changes occur during lifespan. Evidence exists that older participants exhibit altered patterns of functional activation during cognitive tasks. The elderly engage brain areas (especially frontal areas) to a greater extent than young adults; this is most likely to compensate for impaired function in other brain areas (Posterior-Anterior Shift with Aging, PASA; Grady et al., 1994; Davis et al., 2008; Grady, 2012). In contrast to task-based methods, the task-independent approach of a resting-state analysis is appealing due to its ability to assess altered brain function independent of the participant’s active involvement and task understanding as well as independent of their sensory performance. The resting-state approach assesses altered brain functions caused by the summation of subtle physiological and pathological changes across the lifespan, which, in turn, can be linked to respective structural changes as noted above (Reetz et al., 2012). Given the extensive changes of perceptual and cognitive processes and the underlying structural and functional brain changes during healthy aging, it seems reasonable that multisensory integration performance as well is altered throughout the lifespan.

Research on multisensory integration in aging in relation to young adults mainly focused on visual-auditory paradigms and employed mostly static stimuli. These early studies indicated that older adults, when compared to younger adults, do not benefit from multisensory conditions (Stine et al., 1990; Walden et al., 1993; Sommers et al., 2005) or even report a suppressed cortical multisensory integration response in the elderly (Stephen et al., 2010). More recent studies, however, point towards an enhancement of multisensory integration effects also in older adults. Among others, shorter reaction times in response to multisensory events have been reported (Helfer, 1998; Laurienti et al., 2006; Diederich et al., 2008; Mahoney et al., 2011, 2012; Diaconescu et al., 2013). A potential reason for the earlier negative results and more recent positive results can be that the early studies focused on very complex auditory-visual speech perception and thus involve sensory as well as higher-order cognitive processes whereas later studies mostly used simpler combinations of stimuli originating from objects. Another explanation for the conflicting results is the use of different multisensory testing and data analysis techniques. It is also possible that the basic principles for a successful multisensory integration (temporal and spatial accordance, inverse effectiveness, semantic congruency) have been violated within the earlier studies.

Different aspects were discussed as basis for this improvement in multisensory integrative function in elder adults (Mozolic et al., 2012). General sensorimotor and cognitive slowing during aging is obviously not able to explain response times acceleration (Laurienti et al., 2006; Peiffer et al., 2007). However, it was recently demonstrated that older adults have a broader time window of integration as a consequence of increased response times and a wider distribution or range of response times; the combined effect aids older adults to separate stimuli in time (Diederich et al., 2008). In this study, older adults demonstrated a lower probability of integration due to the broader time window; however, in case of a successful integration the gain of older adults was larger compared to younger adults. Age-dependent deficits in top-down selective attention to incoming sensory information do not provide an explanation for the enhancement of multisensory integration in older subjects (Mozolic et al., 2008; Hugenschmidt et al., 2009). One plausible explanation is the principle of inverse effectiveness, i.e., that reduced sensitivity in the individual sensory systems (e.g., rigidity of the lenses, loss of hair cells in the ear and olfactory receptors) combined with age-related alterations in cognitive processing increases the relative magnitude of multisensory enhancements (Hairston et al., 2003). Thereby, multisensory integration becomes more important during aging as it helps to counteract the often-destructive consequences of unisensory deterioration. Mozolic et al. (2012) recently proposed a second possible explanation. They suggested that elderly do not adequately filter sensory noise and hence are more prone to distraction than their younger counterparts. As soon as the extraneous sensory information becomes relevant, however, older adults benefit from this enhanced processing of sensory background information. Evidence for a higher level of background sensory processing in the elderly was also provided by several resting-state studies pointing towards an increased default mode network (DMN) activity (Grady et al., 2006; Li et al., 2007). Furthermore, in elderly, the observed decrease in visual memory and visuo-constructive functions seems to be strongly associated with an age-dependent increase of functional connectivity specifically in the temporal lobe (Schlee et al., 2012).

Age-related changes during information processing (compensatory reallocation, neural compensation, dedifferentiation, inhibition) are based upon basic circuitry of the sensory systems involving several interactive neuronal loops such as prefronto-thalamo-cortical gating between the thalamus and the neocortex in order to effectively process sensory and higher-order cognitive information (Mahoney et al., 2011). Using magnetoencephalography, Diaconescu et al. (2013) indicated that sensory-specific regions showed an increased activity after visual-auditory stimulation in young and old participants, whereas inferior parietal and medial prefrontal areas responded preferentially in older subjects. Further, activation of the latter areas was related to faster detection of multisensory stimuli. This relation was mediated by age-related reductions in gray matter volume in those regions (Diaconescu et al., 2013). The authors propose that posterior parietal and medial prefrontal activity is the basis for the integrated response in older adults. This hypothesis is supported by the theory of PASA described above as well as the theory of cortical dedifferentiation stating that healthy aging is accompanied by decreased specificity of neurons in the prefrontal cortex (Park and Reuter-Lorenz, 2009).

Thus, the neural network governing multisensory integration displays clear age-dependent alterations and age-related changes in cognitive function have clear implications for multisensory processing. That said, although age is the number one major risk factor for a large range of degenerative diseases, it remains to be determined how multisensory integration is affected in different states of age-related diseases, and in particular, diseases of neurodegenerative nature. Unfortunately, to date, there are few published studies on this topic. Any potential changes due to neurodegenerative states might, however, be highly relevant as most of the age-related neurodegenerative disorders are preceded by long presymptomatic periods. Neuropathobiological changes occur many years, even decades, before the clinical manifestation of the disease and numerous compensatory mechanisms and neuroplastic capacities of the human brain remain to be clarified.

The most frequent age-related neurodegenerative disorder is Alzheimer’s disease (AD). Due to aging of populations in both developed and developing societies, AD affects 24.3 million people worldwide and has become one of the most severe socio-economical and medical burdens (Ferri et al., 2005). AD, the most common form of dementia, is a complex disease characterized by an accumulation of β-amyloid plaques and neurofibrillary tangles composed of tau amyloid fibrils. These changes are associated with synapse loss and neurodegeneration leading to a general and progressive loss of cognitive functions, initially predominantly manifested as memory impairment. As studies have revealed that increased DMN activity in healthy aging is associated with a higher level of background sensory processing (Grady et al., 2006; Li et al., 2007), functional changes in AD are of particular interest. Recent studies have mainly demonstrated task-induced deactivations of the DMN as well as a decreased DMN functional connectivity along a continuum from normal aging to pathological conditions (Andrews-Hanna et al., 2007; Hafkemeijer et al., 2012). Resting state functional MRI demonstrated that the regional coherence of brain activity is significantly altered in patients with AD [e.g., Filippi and Agosta, 2011]. Furthermore, abnormalities in the precuneus of patients with amnestic mild cognitive impairment compared to controls were found while AD patients showed alterations of large-scale functional brain networks extending well beyond the DMN. Moreover, episodic-memory related task-based functional neuroimaging studies in AD have revealed increased activity in the precuneus (Browndyke et al., 2013); an area which plays a crucial role in the DMN and has the highest metabolic and blood perfusion rates during resting conditions (Gusnard and Raichle, 2001). The clinical relevance of resting state networks beyond the DMN is notable because the degree of connectivity in the resting state networks may predict individual cognitive and emotional functions (Wang et al., 2010a). Specifically, interhemispheric functional connectivity of the hippocampi (Wang et al., 2010b), as well as connectivity between the hippocampus and the posteriomedial cortices, predicts memory performance in healthy individuals (Wang et al., 2010a). The available research demonstrates that suppression of the DMN during task performance is important. However, conceptualizing the network in terms of suppression implies that the DMN is a sort of nuisance network where its importance to voluntary cognitive functions lies primarily in minimizing its activity during tasks. Consequently, patients with AD that demonstrate hypometabolism in the precuneus/posterior cingulate component of the DMN (Bradley et al., 2002; Chetelat et al., 2008; Langbaum et al., 2009; Petrie et al., 2009; Schroeter et al., 2009) should have very good cognitive functioning during purposeful tasks because the DMN is not active. However, this is clearly not the case. Therefore, further studies with a focus on functional connectivity analyses might foster our understanding of changes in the context of multisensory integration in healthy populations and pathological conditions.

Overall, very little is known about behavioral benefits during multisensory integration in patients with age-related neurodegenerative disorders. Using bimodal stimulation (audio-visual speech presentation), patients demonstrate a limited ability to benefit from concurrent perceptual and linguistic cues compared to healthy aged subjects (Phillips et al., 2009). Given the fact that we have a poor understanding of multisensory integration in neurodegenerative disorders, further studies regarding behavioral and neuronal substrates of multisensory integration are warranted. The knowledge of mechanisms involved in multisensory integration in neurodegenerative disorders, with AD representing the most common one, is of high value given the fact that multisensory stimulation has an inherently high potential for early intervention and thus, also therapeutic application (Staal et al., 2003).

## Conclusion

Advances are being made in disentangling the multisensory integration mechanisms in elderly, thus encouraging future larger longitudinal studies needed to understand the specific neurobiological and neuropathological basis of multisensory integration in health and disease. The current state of research on multisensory integration in healthy aging and age-related neurodegenerative disorders would greatly benefit from further studies as we aim to understand basic mechanisms and potential compensatory strategies of the human brain that help maintain multisensory integration capabilities during both healthy and pathological aging. Early identification of changes in multisensory integration will help to better inform choice of therapy and aid a personalized approach to clinical treatment. Future studies are warranted to determine the clinical translational value of multisensory integration processes in the elderly.

## Conflict of interest statement

The authors declare that the research was conducted in the absence of any commercial or financial relationships that could be construed as a potential conflict of interest.
